# SARN: Shifted Attention Regression Network for 3D Hand Pose Estimation

**DOI:** 10.3390/bioengineering10020126

**Published:** 2023-01-17

**Authors:** Chenfei Zhu, Boce Hu, Jiawei Chen, Xupeng Ai, Sunil K. Agrawal

**Affiliations:** 1 Department of Mechanical Engineering, Columbia University, New York, NY 10027, USA; 2 Department of Rehabilitation Medicine, Columbia University, New York, NY 10027, USA

**Keywords:** hand pose estimation, finger tapping test, hand movement recognition, deep learning, computer vision, depth camera

## Abstract

Hand pose estimation (HPE) plays an important role during the functional assessment of the hand and in potential rehabilitation. It is a challenge to predict the pose of the hand conveniently and accurately during functional tasks, and this limits the application of HPE. In this paper, we propose a novel architecture of a shifted attention regression network (SARN) to perform HPE. Given a depth image, SARN first predicts the spatial relationships between points in the depth image and a group of hand keypoints that determine the pose of the hand. Then, SARN uses these spatial relationships to infer the 3D position of each hand keypoint. To verify the effectiveness of the proposed method, we conducted experiments on three open-source datasets of 3D hand poses: NYU, ICVL, and MSRA. The proposed method achieved state-of-the-art performance with 7.32 mm, 5.91 mm, and 7.17 mm of mean error at the hand keypoints, i.e., mean Euclidean distance between the predicted and ground-truth hand keypoint positions. Additionally, to test the feasibility of SARN in hand movement recognition, a hand movement dataset of 26K depth images from 17 healthy subjects was constructed based on the finger tapping test, an important component of neurological exams administered to Parkinson’s patients. Each image was annotated with the tips of the index finger and the thumb. For this dataset, the proposed method achieved a mean error of 2.99 mm at the hand keypoints and comparable performance on three task-specific metrics: the distance, velocity, and acceleration of the relative movement of the two fingertips. Results on the open-source datasets demonstrated the effectiveness of the proposed method, and results on our finger tapping dataset validated its potential for applications in functional task characterization.

## 1. Introduction

Hand pose estimation (HPE) is an important research topic that is widely studied and applied in many fields, including augmented reality (AR) and virtual reality (VR) [1], human-computer interactions (HCI) [2], robotics [3], and medicine [4,5,6,7,8,9,10,11]. HPE is usually achieved by predicting the positions of a group of hand keypoints that determine the pose of the hand, such as the finger joint center and center of the palm. In medicine, HPE is essential to recognizing hand movement [7,12] and in rehabilitating hand functions [5,8]. It can provide clinicians and physical therapists with accurate estimates of hand movement in different hand tests while maintaining inter-observer reliability [7]. Traditional HPE methods track hand movements using wearable sensors [4,5,6]. Sensor-based methods can track hand positions accurately, but the size and mass of the sensors may interfere with the movement of the hand, leading to measurement errors [7]. Moreover, because of the specifics of the sensors and their calibration, sensor-based methods become limited to specific testing environments.

In recent years, deep learning methods have been used in HPE. Generally, learning-based methods use camera images as input and enjoy two main advantages compared to sensor-based methods. First, subjects can execute hand movements without interference from wearable sensors. Second, hand movements can be performed where images can be acquired. For example, a participant can do the test at home and record it in a video. This can also provide safety to the participant and the caregiver in scenarios where there are risks of potential infection. Based on these, learning-based methods are gradually gaining popularity. Some studies have implemented learning-based HPE on RGB images for hand movement recognition and functional rehabilitation [12,13].

**Figure 1:**
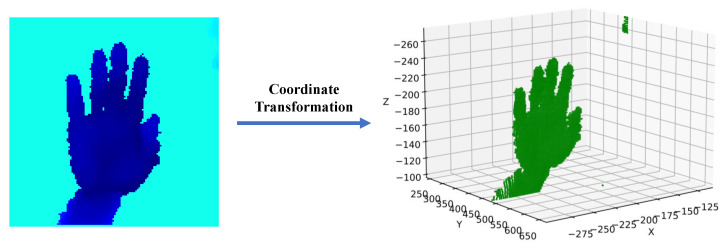
**Left**: a colorized depth image of a hand. Each pixel on the depth image has its position in the image coordinate frame, which is determined by the row and column in which the pixel is located, and the depth value from the camera. **Right**: the positions of all the points corresponding to the pixels on the depth image in the 3D world coordinate frame centered at the depth camera. The coordinates of pixels in the two coordinate frames can be converted to each other by coordinate transformation. In the following sections, we use pixels to refer to points in 3D space.

However, the accuracy of RGB-image-based methods is much lower than sensor-based methods because RGB images lack spatial information. In contrast, depth images additionally have distance information associated with each pixel in the image with respect to a camera. Depth images are 2.5D images; each pixel in the image with a non-zero depth value corresponds to a point on the surface of the object, as shown in Figure 1. Depth-image-based methods [14,15,16,17,18,19,20] can perform better than RGB-image-based methods in hand movement recognition. However, these methods are still limited in recognizing hand posture accurately.

In order to improve the accuracy of HPE, in this paper, we propose a novel architecture of a shifted attention regression network (SARN) to perform convenient and accurate hand pose estimation. Given a depth image, SARN predicts the position of hand keypoints in two stages. In each stage, the proposed model predicts the spatial relationships between pixels and hand keypoints through a backbone network and a dense extraction module. The two stages are stacked in a cascade form by a soft input aggregation module, where the second stage of the network refines the predictions after the first stage. At the end of the second stage, the proposed model utilizes the spatial relationships pixel-wise to obtain the estimation of the position of each hand keypoint.

To test the effectiveness of the proposed method, we first conducted experiments on three open-source datasets of 3D hand poses, NYU [21], ICVL [22], and MSRA [23]. These datasets are often used as benchmarks for evaluating 3D hand pose estimation methods [14,15,16,17,18,19,20]. On all these three datasets, SARN achieved state-of-the-art performance. To validate the feasibility of the proposed method in hand movement recognition, we constructed our own dataset with depth images, which we call the PAKH dataset. This dataset comprises 26K depth images from 17 healthy subjects based on the finger tapping test, an important component of neurological examinations administered to Parkinson’s patients. Each depth image is annotated with the tips of the thumb and index finger.

Overall, the contributions of this manuscript include:

- We propose a novel depth-image-based method, SARN, for convenient and accurate 3D hand pose estimation during functional tasks.
- We propose a novel structure, soft input aggregation, to connect a multi-stage model to reduce the error of 3D hand pose estimation.
- We construct a dataset consisting of 26K depth images from 17 healthy subjects based on the finger tapping test, often used in neurological examinations of Parkinson’s patients.
- For PAKH, the proposed method achieved a mean error of 2.99 mm for the hand keypoints and comparable performance on three task-specific metrics: the distance, velocity, and acceleration of the relative movement of the two fingertips.

## 2. Related Work

### 2.1. Sensor-Based HPE Methods

Sensors are currently the primary tools for performing HPE in hand movement recognition. A popular way to implement sensor-based HPE is by sensor gloves. Pei-Chi et al. [4] designed a data glove embedded with 9-axis inertial sensors and force-sensitive resistors to enable hand pose recognition in real time. Yang et al. [5] developed a sensor glove based on resistive bend sensors to monitor finger joint angles. Moreira [8] designed a glove with eleven inertial measurement units attached to the proximal and distal phalanges and the back of the hand to capture hand movements. Other studies have used sensors attached to the hand to determine its pose. Chen et al. [6] designed a multi-point tracking system using electromagnets and magnetic sensors to track fingertip movements in real time. Julien et al. [10] used a triaxial accelerometer mounted at the fingertip to extract hand movement features. Ji-Won et al. [11] used a miniature lightweight gyrosensor to measure finger taps. Yuko et al. [9] evaluated hand movement using magnetic sensors fixed on hands. Abraham et al. [24] used lensless smart sensors and designed computational algorithms to track the positions of infrared light-emitting diodes attached to the hands and perform hand gesture recognition. Gosala et al. [25] fused the predictions of a stretch-sensing soft glove, three IMUs, and an RGB-D camera based on the availability and confidence estimation to enable seamless hand tracking. All of these methods have sensors attached to the human hand. While these devices can accurately track the motion of the hand, their size and mass interfere with hand movements, which leads to errors in pose estimation during hand movements.

### 2.2. Learning-Based HPE Methods

Learning-based methods can be classified as regression-based methods and detection-based methods. Regression-based methods directly predict hand keypoint positions based on a depth image. Oberweger et al. [18] improved the predictions of hand poses by introducing a prior distribution of hand keypoints within a convolutional neural network (CNN). Later, they [14] refined their model by making simple improvements such as better initial hand localization. Chen et al. [15] reorganized the features for 3D HPE by dividing feature maps into several sub-regions based on the initial estimate. Ge et al. [26] proposed to directly process the 3D points that model the visible surface of the hand for pose regression and designed another network to refine the fingertip location. Chen et al. [20] used a subnetwork to assign semantic labels for each point and used another network to integrate the semantic priors with both input and late fusion strategy and regress the final hand pose.

In contrast, detection-based methods predict dense representations. A dense representation refers to the spatial relationships between pixels and hand keypoints. Dense representations can be classified as heatmaps and offset maps. A heatmap represents the likelihood of a hand keypoint appearing at the position of each pixel, and an offset map consists of the vectors pointing from a hand keypoint to each pixel. Both heatmaps and offset maps can be 2D or 3D, depending on whether they are calculated in the image coordinate frame or in the world coordinate frame. A detection-based model uses the dense representation predicted to infer the position of hand keypoints. Ren et al. [27] used a 3D offset map to unify the predictions in three directions and derived the hand keypoint positions by post-processing methods. Moon et al. [28] estimated a 3D heatmap for each hand keypoint by implementing a voxel-to-voxel prediction based on 3D grids. Some studies integrated the estimation of hand keypoint positions into the pipeline to reduce the gap between training the network and inferring the hand keypoint positions. Fu et al. [29] used a 2D offset map to integrate the 2D positions of the hand keypoints in the image and used another branch to predict their depth values. Mohammad et al. [17] predicted a 2D heatmap to integrate the 2D positions of hand keypoints and supervise the estimation of depth values of each hand keypoint. Huang et al. [30] predicted the hand keypoint positions by weighting the 3D offset map with a 3D heatmap. The dense representations proposed by these methods did not take full advantage of the spatial information contained in the depth map, and this limits the performance of these methods.

The proposed method leverages a fused heatmap for estimating hand keypoint positions similar to [17], which is one of the state-of-the-art methods for 3D HPE. However, our implementation of the heatmap has several features that make it unique and enable it to perform better than previous works. First, our heatmap is 3D instead of 2D. Second, we perform the estimation of the hand keypoint positions as a whole through the 3D offset map rather than separating it into estimating the 2D position in the image and depth values. Moreover, our training process is supervised by the ground truth of a 3D heatmap instead of 2D positions in the image. With these features, our method can better utilize the spatial information in the depth map and thus achieve higher precision.

## 3. Materials and Methods

### 3.1. Overview of the Framework

**Figure 2:**
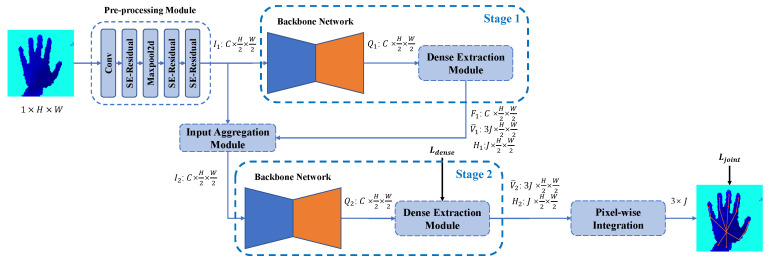
The working pipeline of the proposed SARN. The input of SARN is a depth image of size 1×H×W, and the output is the 3D position of *J* hand keypoints, which has a size of 3×J. *H* and *W* represent the height and weight of the input depth image. Pre-processing module extracts *C* feature maps of size $\frac{H}{2}\times \frac{W}{2}$ from the input depth image. SE-Residual is a variant of the residual block [31]; we will introduce its structure in Section 3.3. $I_{i}$ and $Q_{i}$ stand for the input features and output of the backbone network at stage *i*. ${\bar{V}}_{i}$ and $H_{i}$ denote the dense representation predicted by the dense extraction module at stage *i*. For $I_{i}$, $Q_{i}$, ${\bar{V}}_{i}$, and $H_{i}$, i=1,2. $F_{1}$ is an intermediate feature in the dense extraction module at the first stage.

The working pipeline of the proposed SARN is shown in Figure 2. SARN is a two-stage model, which consists of a pre-processing module, an input aggregation module, two backbone networks, two dense extraction modules, and a pixel-wise integration module. Each stage of SARN consists of a backbone network and a dense extraction module. Both stages extract a dense representation of the spatial relationships between pixels and hand keypoints in the world coordinate frame. The input aggregation module extracts feature maps from the first stage and transfers them into the second stage to connect the two stages. The pixel-wise integration module takes the dense representation output from the second stage to infer the 3D position of each hand keypoint. We implement supervision on both the dense representation of the second stage and the hand keypoint position. Each part is described in detail in the following sections.

### 3.2. Dense Extraction Module

**Figure 3:**
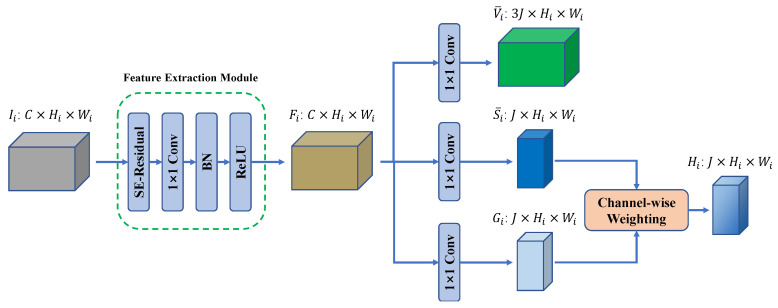
Illustration of the dense extraction module at stage *i*. The feature extraction module consists of a SE-Residual block, a Conv layer, a batch normalization (BN) layer, and a ReLU activation function. ${\bar{V}}_{i}$ and ${\bar{S}}_{i}$ are the two parts of the predicted 3D offset map, and ${\bar{S}}_{i}$ and $G_{i}$ form the shifted attention heatmap. $C_{i}$, $H_{i}$, and $W_{i}$ are the number, height, and width of the feature maps at stage *i*. In our implementation, $C_{i}=C$, $H_{i}=\frac{H}{2}$, and $W_{i}=\frac{W}{2}$ for i=1,2.

Figure 3 shows the components of the dense extraction module at the *i*-th stage. A dense extraction module consists of a feature extraction module, three parallel Conv layers, and a channel-wise weighting layer. The dense extraction module uses the feature maps output by the backbone network at the same stage to predict a dense representation of spatial relationships between pixels and hand keypoints, which consists of a 3D offset map and a characteristic shifted attention heatmap.

#### 3.2.1. 3D Offset Map

A 3D offset is a vector pointing from the position of a hand keypoint to the position of a pixel in the world coordinate frame. The 3D offset map of a hand keypoint consists of 3D offsets pointing from that hand keypoint to all pixels in the depth image. The 3D offset map represents the spatial relationships between a hand keypoint and each pixel. Unlike a 2D offset map or heatmap, the 3D offset map keeps the original representation of the depth image and can better utilize the spatial information in it [32]. Based on this, we chose the 3D offset map as the dense representation of the spatial relationship between pixels and hand keypoints. For a hand keypoint *j*, a depth image of size $1\times H_{d}\times W_{d}$ can generate $H_{d}\times W_{d}$ 3D offsets, so the 3D offset map of keypoint *j*, $O^{j}$, is of size $3\times H_{d}\times W_{d}$. The formulation of a 3D offset in $O^{j}$ is shown below.

(1) $$O^{j}\left(p\right)=\left\{\begin{array}{cc} p-p_{j} & ∥p-p_{j}{∥}_{2}\leq \theta \\ 0 & \mathrm{otherwise} \end{array}\right.$$

where $O^{j}\left(p\right)\in R^{3}$ represents the 3D offset pointing from hand keypoint *j* to pixel p, $p\in R^{3}$ denotes the position of a pixel in the world coordinate frame, and $p_{j}\in R^{3}$ denotes the ground-truth position of hand keypoint *j*. θ stands for the radius of a sphere that is centered at the position of keypoint *j* to indicate the space where candidate supporting points are located. Those candidate supporting points are spatially close pixels of keypoint *j*. If p is not a supporting point of keypoint *j*, that is, if the Euclidean distance between pixel p and keypoint *j* is larger than θ, the 3D offset between them will be set to zero. The 3D offset map $O^{j}$ can be further decomposed into a spatial closeness heatmap $S^{j}$ of size $1\times H_{d}\times W_{d}$ and a directional unit vector map $V^{j}$ of size $3\times H_{d}\times W_{d}$. This decomposition is achieved by decomposing each 3D offset in $O^{j}$ into a spatial closeness and a directional unit vector, as follows:

(2) $$S^{j}\left(p\right)=\left\{\begin{array}{cc} \frac{\theta -∥p-p_{j}{∥}_{2}}{\theta } & ∥p-p_{j}{∥}_{2}\leq \theta \\ 0 & \mathrm{otherwise} \end{array}\right.$$

(3) $$V^{j}\left(p\right)=\left\{\begin{array}{cc} \frac{p-p_{j}}{∥p-p_{j}{∥}_{2}} & ∥p-p_{j}{∥}_{2}\leq \theta \\ 0 & \mathrm{otherwise} \end{array}\right.$$

where $S^{j}\left(p\right)\in R$ represents the spatial closeness between hand keypoint *j* and pixel p. $V^{j}\left(p\right)\in R^{3}$ denotes the directional unit vector pointing from hand keypoint *j* to pixel p.

For each keypoint *j*, we calculate a spatial closeness heatmap $S^{j}$ and a directional unit vector map $V^{j}$. *J* hand keypoints result in *J* spatial closeness heatmaps and *J* directional unit vector maps. We stack the spatial closeness heatmaps of all keypoints to obtain an overall spatial closeness heatmap *S* and the stack directional unit vector maps of all keypoints to obtain an overall directional unit vector map *V*; that is:

(4) $$S=\left(S^{1},S^{2},...S^{J}\right),\;\;V=\left(V^{1},V^{2},...V^{J}\right)$$

where *S* is of size $J\times H_{d}\times W_{d}$, and *V* is of size $3J\times H_{d}\times W_{d}$.

In our implementation, we interpolate the input depth image to obtain a smaller depth image of size $1\times \frac{H}{2}\times \frac{W}{2}$ and then use this new depth image to calculate the 3D offset map, so $H_{d}=\frac{H}{2}$ and $W_{d}=\frac{W}{2}$. In the dense extraction module, we use two branches to predict *S* and *V* separately; this process is supervised by the ground-truth value computed by the above formulas.

#### 3.2.2. Shifted Attention Heatmap

**Figure 4:**
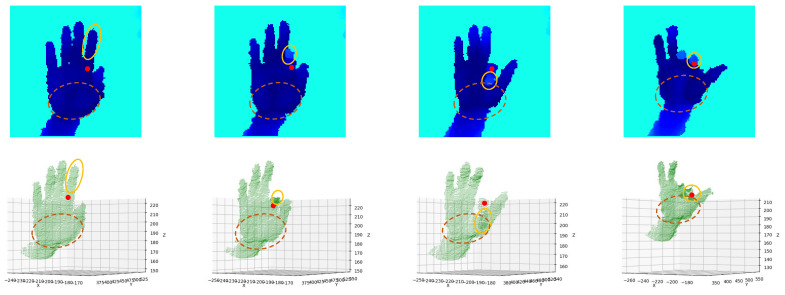
Qualitative results of the spatial correlations between the MCP joint of the index finger and different parts of the hand. The red point denotes the center of the MCP joint of the index finger. The solid orange boundary indicates the area of the index finger. The brown dashed boundary indicates the area of the palm.

The spatial closeness heatmap *S* is calculated by the distances between pixels on the depth image and hand keypoints; it can be seen as a natural representation of spatial correlations between pixels and keypoints [30]. When a pixel is closer to a hand keypoint in space, the spatial correlation between them is usually stronger. This pixel can thus provide more information when predicting the position of that hand keypoint. Former works, therefore, weighted the 3D offset map with a spatial closeness heatmap [30] or implemented post-processing methods such as argmax on it [27] to infer the hand keypoint’s position. However, spatial closeness does not always correspond exactly to spatial correlation. This can be explained by hand geometry. Human hands consist of many parts, some of which are more flexible than others. For example, the fingers are more flexible than the palm. When bending a finger, these points on the tip or the proximal interphalangeal (PIP) joint of that finger may move significantly, with the metacarpophalangeal (MCP) joint keeping its position, as shown in Figure 4. In this case, though spatially close to the MCP joint, points on the finger do not have strong spatial correlations with it.

In contrast, the motion of the palm is much simpler. Without moving the MCP joint of the index finger, the positions of the points on the palm can hardly change in a complex way. Therefore, a point on the palm can have a stronger spatial correlation with the MCP joint of the index finger, even though they are farther from it compared to those points on the finger.

To better utilize such implicit spatial correlations that are difficult to quantify, we use another branch to learn a heatmap without the supervision of the ground truth of the spatial closeness heatmap. Unlike the spatial closeness heatmap, this heatmap aims to find geometrically meaningful points, so we name it the geometry closeness heatmap. Then, we fuse the predicted spatial closeness heatmap and geometry closeness heatmap to leverage the information contained in both. This fusion operation is achieved by a channel-wise weighting of the above two heatmaps, as follows:

(5) $$H_{i}=\alpha_{i}{\bar{S}}_{i}+\left(1-\alpha_{i}\right)G_{i}$$

where $\alpha_{i}\in R^{J}$ denotes a learnable channel-wise weighting factor at stage *i*, and $H_{i}=\left(H_{i}^{1},H_{i}^{2},...H_{i}^{J}\right)$ and $G_{i}=\left(G_{i}^{1},G_{i}^{2},...G_{i}^{J}\right)$ represent the fused heatmap and the geometry closeness heatmap at stage *i*. ${\bar{S}}_{i}=\left({\bar{S}}_{i}^{1},{\bar{S}}_{i}^{2},...{\bar{S}}_{i}^{J}\right)$ represents the predicted spatial closeness heatmap at stage *i*. At the end of the second stage, we obtain the prediction of each pixel for the position of each hand keypoint by combining the position of each pixel with the predicted 3D offset map of each keypoint. After that, we use the shifted attention heatmap to weight the predictions of all pixels to obtain the final estimation of the position of each hand keypoint. For example, the estimated position of hand keypoint *j* is:

(6) $${\bar{p}}_{j}=\sum_{p}\left(\left(\theta {\bar{S}}_{2}^{j}\left(p\right)-\theta \right){\bar{V}}_{2}^{j}\left(p\right)+p\right)H_{2}^{j}\left(p\right)$$

in which ${\bar{p}}_{j}$ is the estimated position of keypoint *j*. ${\bar{S}}_{2}^{j}$ and ${\bar{V}}_{2}^{j}$ are the predicted spatial closeness heatmap and directional unit vector map of keypoint *j* at the second stage. $H_{2}^{j}$ is the shifted attention heatmap of keypoint *j* at the second stage, which is the *j*-th channel of $H_{2}$.

**Figure 5:**
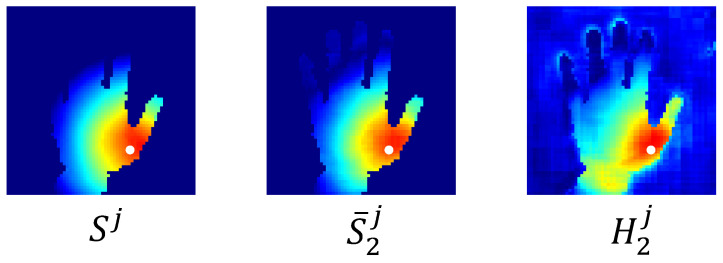
Qualitative results of the heatmaps of the MCP joint center of the thumb. The white dot represents the center of the MCP joint of the thumb. Warm-colored areas are considered more spatially correlated with the MCP joint than cold-colored areas. Compared to the spatial closeness heatmap, the fused heatmap finds more spatially correlated points on the wrist.

Figure 5 shows a qualitative result of the spatial closeness heatmap and the fused heatmap of the center of the MCP joint of the thumb. The spatial closeness heatmap calculates spatial correlations based entirely on spatial proximity; pixels closer to the MCP joint of the thumb are therefore considered more important. In contrast, the fused heatmap finds some geometrically important points on the wrist while also focusing on the neighborhoods of the MCP joint of the thumb. We refer to the transformation from the spatial closeness heatmap to the fused heatmap as attention shifting and call the fused heatmap a shifted attention heatmap. The difference between the two heatmaps caters to our intuition: though far away in space, some geometrically meaningful points may be of greater spatial correlation with hand keypoints than those spatial proximity points.

### 3.3. Backbone Network

**Figure 6:**
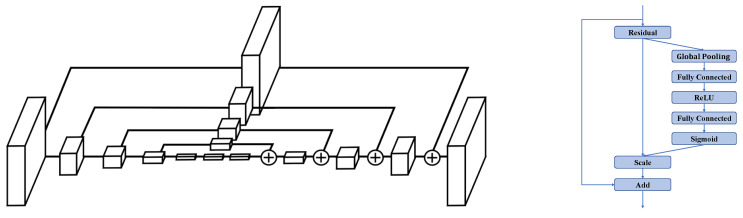
Illustration of the backbone network. **Left**: the structure of the Hourglass network [33]. Each box represents a residual block. **Right**: the structure of the SE-residual block. In SE-Hourglass, residual blocks are replaced by SE-residual blocks.

We use the SE-Hourglass network, a simple modification of Hourglass [33], as our backbone network. Hourglass is an encoder-decoder network that uses a bottom-up, top-down design, combined with skip connections, to extract features of various scales. Several hourglass networks can be stacked together to repeat this process; while the first stage outputs a bad prediction, subsequent stages reevaluate and refine it to achieve better performance. We obtain the SE-Hourglass network by replacing the residual blocks used in Hourglass with SE-residual blocks. SE-residual is a variant of the residual block with a squeeze-and-excitation (SE) module. SE-residual can better utilize the feature information by exploring the inter-dependencies between channels [34], therefore performing better than the original block. The structure of the Hourglass network and the SE-residual block are shown in Figure 6.

### 3.4. Soft Input Aggregation

**Figure 7:**
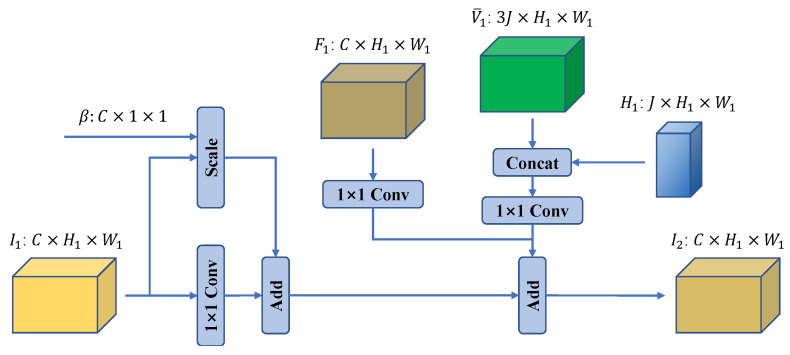
Structure of the proposed soft input aggregation module. β represents the learnable channel-wise factor. Concat denotes the concatenate operation. Inputs of the proposed soft input aggregation module are $I_{1}$, $F_{1}$, ${\bar{V}}_{1}$, and $H_{1}$. Output is $I_{2}$, the input features of the second stage.

**Figure 8:**
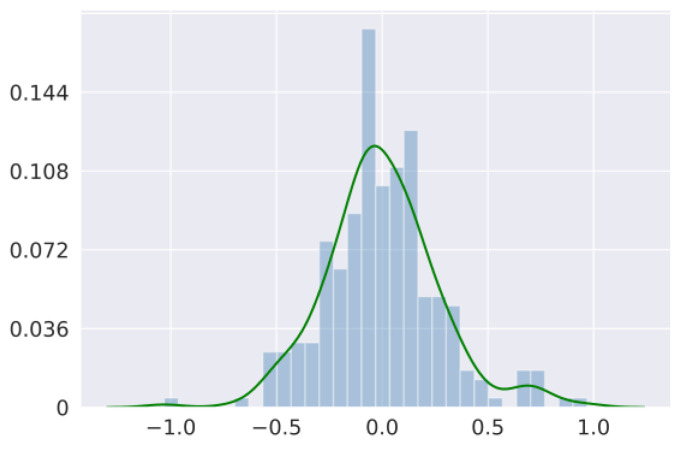
Qualitative result of the distribution of the channel-wise factor β.

Following former pose estimation works [30,32,35,36], we stack two stages for better estimations. In previous works, multiple stages were connected sequentially [32,36] or stacked together by Conv layers [35]. These methods cannot transfer information between stages efficiently and therefore fail to utilize the advantages of the multi-stage model. To solve this problem, we propose a novel soft input aggregation method to better transfer information between stages. The structure of the proposed soft input aggregation module is shown in Figure 7. The proposed soft input aggregation module uses three parallel Conv layers to extract feature maps from three levels of the first stage: input, intermediate, and output. It also scales the input of the first stage with a learnable channel-wise factor β. Then, it adds together all the extracted feature maps and the scaled input and transfers them to the second stage. The most important part of this module is the learnable channel-wise factor β used to scale the input of the first stage. It is inspired by the work [37], which claimed that the presence of identity connections in blocks such as residuals might have undesirable effects. While their work focuses on improving residual modules, our approach aims at transferring information between different stages in a stacked model. Figure 8 shows the resulting distribution of β normalizing to [−1,1] after training. Clearly, this learnable channel-wise factor approximates a normal distribution, with some channels having positive and some having negative weights.

### 3.5. Loss Function Design

Following [30], we implement supervision on both dense representation and hand keypoint positions. We use the smooth L1 loss [27] as the loss function for both supervisions, that is:

(7) $$L_{dense}=smooth_{L_{1}}\left(S-{\bar{S}}_{2}\right)+smooth_{L_{1}}\left(V-{\bar{V}}_{2}\right)$$

(8) $$L_{coord}=\sum_{j=1}^{J}smooth_{L_{1}}\left(p_{j}-{\bar{p}}_{j}\right)$$

where $L_{dense}$ denotes the loss of dense representation and $L_{coord}$ denotes the loss of hand keypoints positions. ${\bar{V}}_{2}=\left({\bar{V}}_{2}^{1},{\bar{V}}_{2}^{2},...{\bar{V}}_{2}^{J}\right)$ represents the predicted unit vector map at the second stage. The total loss is a weighting of the two losses, which can be represented as:

(9) $$L=\sigma L_{dense}+\left(1-\sigma \right)L_{coord}$$

where σ is a parameter that controls the weights of the two losses, which is set to 0.5 in our implementation.

### 3.6. Implementation Details

Our method was implemented with PyTorch using the Adam optimizer with an initial learning rate of 0.001. The batch size was set to 20. We multiplied the learning by 0.7 when the loss was not decreasing in 3 steps. Following former works, for both training and testing phases, we first used a pre-trained CNN network [27] to obtain the hand center and extract the hand region from a depth image, crop and resize it to the fixed size of 128×128, and normalize depth values to [−1,1]. For the training phase, data augmentation was applied by geometric transformations including in-plane rotation ([−180,180]), 3D scaling ([0.9, 1,1]), and random translation ([−10,10]). SARN was trained on the NYU dataset for 25 epochs, on the MSRA dataset for 30 epochs, and for 35 epochs on the ICVL dataset and on our PAKH dataset.

## 4. Experiments and Results

### 4.1. Datasets and Evaluation Metrics

We conducted experiments on three open-source 3D hand pose datasets, the NYU dataset [21], the ICVL dataset [22], and the MSRA dataset [23], to test the effectiveness of our method and on our PAKH dataset to validate the feasibility of our method in finger tapping tests.

**NYU Dataset.** The NYU dataset was collected from a frontal view and two side views. Each view of the NYU dataset provides 72K and 8K depth images with 36 hand keypoint annotations for training and testing, respectively. Following the protocol used by [38], we applied our method on only the frontal view with a subset of 14 annotated hand keypoints.

**ICVL Dataset.** The ICVL dataset consists of 22K depth frames for training and 1.6K depth frames in two sequences for testing collected from 10 subjects with 16 hand keypoint annotations. Furthermore, ICVL also provides about 300K augmented training frames by in-plane rotations of the original images.

**MSRA Dataset.** The MSRA dataset contains 76.6K depth images collected from 9 subjects. Each subject performed 17 different hand gestures, and each depth frame was annotated with 21 hand keypoints. Following [23], we adopted the leave-one-subject-out cross-validation strategy for model evaluation on the MSRA dataset.

**Figure 9:**
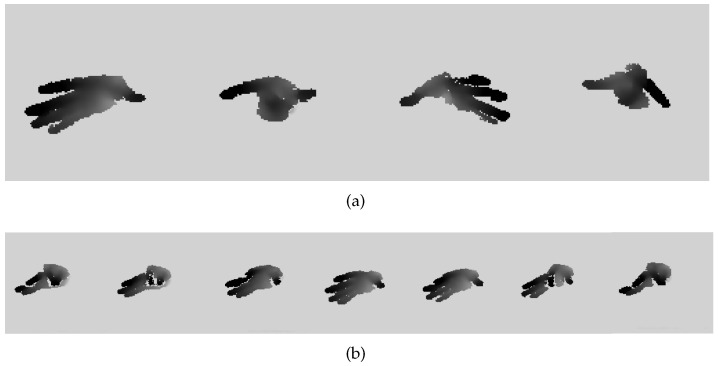
Finger tapping sample frames from PAKH dataset. (**a**) Four hand gestures of finger tapping test in the PAKH dataset, from left to right: right hand with the other three fingers stretched out; right hand with the other three fingers folded onto the palm; left hand with the other three fingers stretched out; left hand with the other three fingers folded onto the palm. (**b**) One complete tapping consisting of an opening phase (frame 1 to 4) and a closing phase (frame 4 to 7).

**Our PAKH Dataset.** The PAKH dataset was constructed based on the finger tapping test, an important component of neurological examinationss administered to Parkinson’s patients proposed by the Movement Disorder Society [39]. During the finger tapping test, the participant is asked to tap the tip of the index finger against the tip of the thumb rapidly while opening the fingers again as far as possible in succession ten times. This test aims to evaluate the severity of Parkinson’s disease in patients by their inability to open and close their fingers repeatedly during the test. In our experiment, each participant was asked to perform the finger tapping test two times with each of the four hand gestures shown in Figure 9a. During the finger tapping test, an Intel RealSense D435i depth camera [40] was placed at the front of the participant to capture hand movements in the form of depth images. Participants were allowed to perform the test at their comfortable positions within the area that can be captured by the camera. Figure 9b shows a sample of a complete tapping. Tips of the thumb and index finger were annotated on depth images to provide the ground truth of the hand keypoint position. Seventeen healthy subjects (age 23.6±1.7 years, height 177.3±5.7 cm, weight 73.1±15.5 kg, BMI 23.1±4.1) participated in our experiments, during which 26K depth images were collected. We split subjects into the training set and test set by the ratio of 13:4. Table 1 shows the number of frames of each participant with different gestures in the training set and test set.

**Evaluation Metrics.** We evaluated the performance of the proposed method on three open-source datasets using two commonly used metrics: per-keypoint and all-keypoint mean error and success rate. The keypoint error is the Euclidean distance between the predicted and ground-truth hand keypoint positions. Per-keypoint and all-keypoint mean error is calculated by averaging the keypoint error of each keypoint and all keypoints over all test frames. The success rate is the percentage of test frames with each keypoint error less than a certain threshold. On the PAKH dataset, we evaluated our method using all-keypoint mean error and several task-specific metrics.

### 4.2. Comparison with State-of-the-Art Methods

We compared our model with state-of-the-art methods on the NYU, ICVL, and MSRA datasets. These methods include regression-based methods: Pose-REN [15], REN-9×6×6 [19], HandPointNet [26], SHPR-Net [20], and 3DCNN [41], as well as detection-based methods: TriHorn-Net [17], Point-to-Point [38], CrossInfoNet [42], V2V [28], and DenseRegression [32].

**Figure 10:**
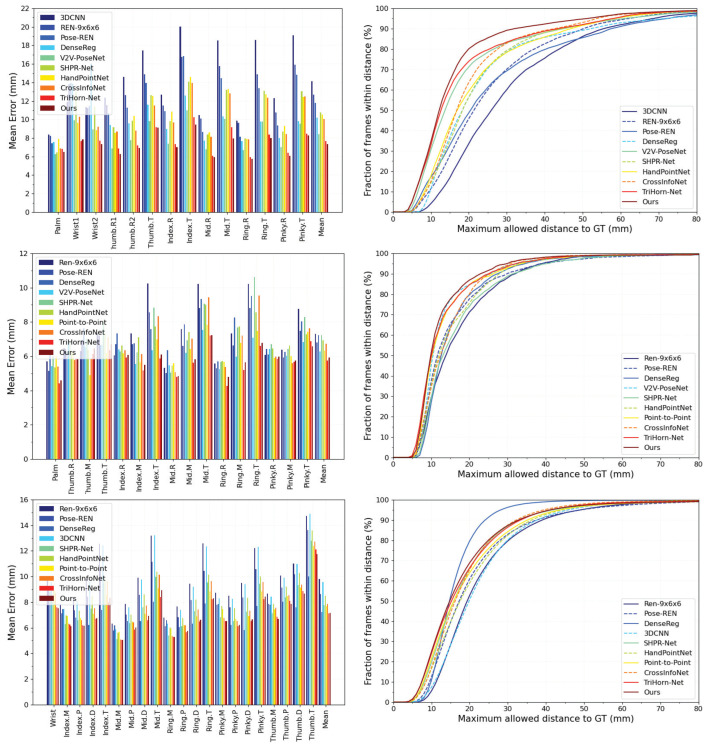
Comparison with state-of-the-art methods on NYU (**top**), ICVL (**middle**), and MSRA (**bottom**) datasets. The all-keypoint and per-keypoint mean errors are shown in the left column, and the success rate over different thresholds is shown in the right column.

Figure 10 shows the per-keypoint and all-keypoint mean error (left column) and success rate (right column). Table 2 summarizes the performance of state-of-the-art methods by the all-keypoint mean error in millimeters. The result indicates that the proposed method outperforms all state-of-the-art methods on the NYU dataset and achieves state-of-the-art performance on the ICVL dataset and MSRA dataset with an all-keypoint mean error of 7.32 mm, 5.91 mm, and 7.17 mm, respectively.

**Figure 11:**
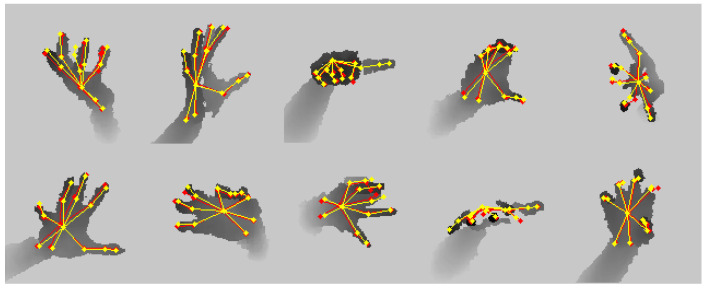
Qualitative results on NYU dataset. Ground truth is shown in red, and the prediction is in yellow.

Figure 11 shows the qualitative result of SARN on the NYU dataset.

### 4.3. Ablation Study

To test the effectiveness of the proposed SARN, we conducted three exploration experiments on the NYU dataset since it is a more general dataset with generous hand gestures.

**Shifted Attention Heatmap.** To analyze the effectiveness of the proposed shifted attention heatmap, we implemented the proposed model with different heatmaps: (1) a spatial closeness heatmap; (2) a geometry closeness heatmap; (3) a shifted attention heatmap (shared weights); (4) a shifted attention heatmap (stage-wise weights). The difference between 3 and 4 is that in 3, two stages of the model share the weights for fusing the two heatmaps, which means $\alpha_{1}=\alpha_{2}$, while in 4, each stage learns its own weights.

Results shown by the all-keypoint mean error in Table 3 indicate that the proposed shifted attention heatmap performs better than both the spatial closeness heatmap and the geometry closeness heatmap, and the shifted attention heatmap with stage-wise weights performs best.

**Backbone Structure.** To investigate the impact of backbone network selection on the performance of the proposed model, we conducted experiments on two commonly used network architectures: Hourglass and ResNet. We implemented the proposed model by taking the ResNet network with different depths (18, 34, and 50), Hourglass, and SE-Hourglass as the backbone network. We also implemented the model with different numbers of stages (1 or 2) to validate the impact of the refinement stage. For the ResNet architecture, we stacked several deconvolution layers after the original out layer to generate the dense representation. In this experiment, we discarded the proposed soft input aggregation module and used only Conv layers to connect stages in two-stage models to reduce its impact.

As shown in Table 4, the performance of ResNet backbones gradually improves as the number of layers increases. ResNet-50 achieves a mean error of 7.69 mm. The Hourglass network performs slightly better than ResNet-34 but worse than ResNet-50. SE-Hourglass achieves a lower error than Hourglass with few extra parameters. Compared to the ResNet architecture, the Hourglass architecture is much smaller regarding the number of parameters. With a refinement stage, both Hourglass or SE-Hourglass backbones perform better than ResNet-50 with a smaller number of parameters, and SE-Hourglass achieves the lowest error.

**Input Aggregation.** In this section, we study the effectiveness of our soft input aggregation module. Here, we focus on the processing of the input of the first stage $I_{1}$. We tested four input aggregation methods, which process $I_{1}$ in different ways: (1) no processing; (2) using a convolution layer to extract feature maps; (3) using a convolution layer to extract feature maps and add them with $I_{1}$; (4) the proposed soft input aggregation method. The difference between 3 and 4 is that in 4, we use a channel-wise factor to scale $I_{1}$. The results shown in Table 5 indicates that for input aggregation methods: (1) raw input $I_{1}$ achieves a lower error than feature maps extracted by a convolution layer; (2) adding the extracted features with $I_{1}$ achieves better accuracy; (3) adding the extracted features with channel-wise scaled $I_{1}$ achieves the best performance.

### 4.4. Performance on Our PAKH Dataset

We used our PAKH dataset to test the feasibility of the proposed model in hand movement recognition. For this dataset, we used the commonly used all-keypoint mean error and some task-specific metrics for model evaluation. Following [12,43], several kinematic features of the tapping were deemed significant for evaluating the severity of the disease. These features included average tapping speed, tapping acceleration, and average opening velocity of the index finger. To fully validate the capabilities of the proposed model on the finger tapping test, we took the distance, velocity, and acceleration of the relative movement between the two fingertips into consideration and introduced distance error (Dis Err), velocity error (Vel Err), and acceleration error (Acc Err). Following [7], the Vel Err and Acc Err are calculated by the finite difference between individual frames of ground truth and predictions while ignoring the sampling interval. Results are shown in Table 6, where Vel Err and Acc Err are computed as aforementioned, and all-keypoint mean error (Pos Err) and Dis Err are in millimeters.

**Figure 12:**
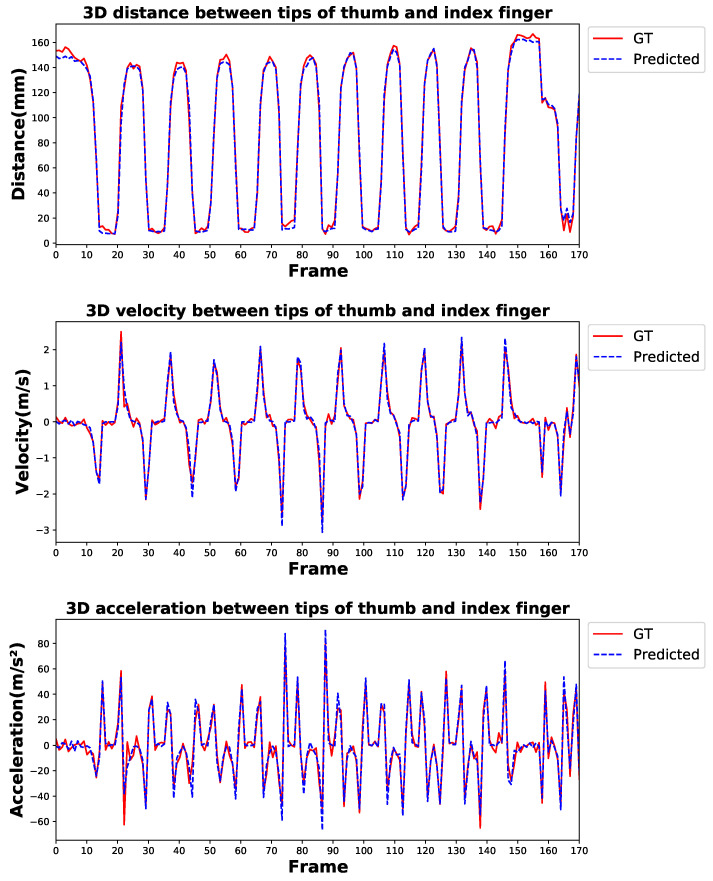
Qualitative result comparison between ground truth and predicted tapping distance, velocity, and acceleration during a trial of the finger tapping test. GT denotes the ground truth.

The results indicate that the proposed method performs well in predicting hand keypoint position with a 2.99 mm all-keypoint mean error while also achieving comparable performance in estimating the distance, velocity, and acceleration of the relative movement between the two fingertips. Figure 12 shows the qualitative result comparison between the ground truth and predicted distance, velocity, and acceleration of the movement during a trial of the finger tapping test, which can serve as an intuitive reflection of the performance of the proposed method.

**Figure 13:**
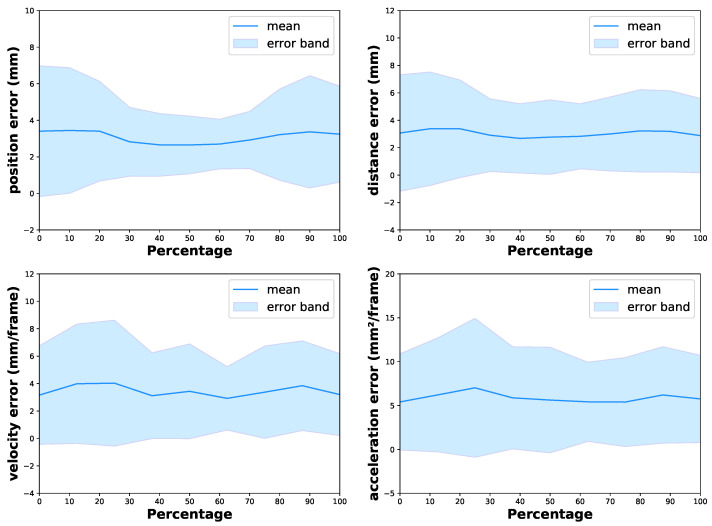
Prediction error bands of SARN during the moving phase.

Moreover, we also analyzed the variation in the performance of the proposed method with respect to different moving states. Specifically, the resting time between trials was removed, and the frames of each tapping in each trial were labeled as percentages from 0 to 100 proportionally, with 0 denoting the beginning of the opening phase, 50 denoting the end of the opening phase, which is also the beginning of the closing phase, and 100 denoting the end of the closing phase. For example, in Figure 9b, frames one to four are 0% → 50%, and frames four to seven are 50% → 100%. Then, we calculated the errors of SARN at different percentages, and the results are shown in Figure 13. For all the evaluation metrics, our model achieves comparable and reasonable error and standard deviation across the whole movement, which indicates that our model is accurate and stable during the entire test. At the beginning and end of the tapping, the position error is slightly larger than those in the middle; this may be attributed to the severe self-occlusion on the fingertips when the two fingers are close to each other, as shown in the first and last frame in Figure 9b.

## 5. Discussion

### 5.1. A Novel Deep Learning Framework for Hand Movement Recognition

In clinical practice, the convenient and accurate assessment of hand poses is critical in hand movement recognition and related neurological examinations. Deep learning has made hand pose estimation easy to perform, but existing learning-based methods are not accurate enough, and their feasibilities in hand movement recognition have rarely been tested. To this end, we proposed a novel network architecture to improve the accuracy of HPE and constructed a hand movement dataset based on a finger tapping test to validate the feasibility of the proposed method in hand movement recognition. Our model estimates the hand keypoint position based on the spatial correlations between different parts of the hand. Inspired by hand geometry, we introduced a novel shifted attention heatmap to leverage both spatial closeness and geometry closeness between different hand parts. Qualitative results and ablation studies have validated the effectiveness of this design. In addition, we also investigated the impact of inter-stage connections on the performance of a multi-stage model. Previous works have widely used multi-stage models to perform HPE, of which only a few have paid attention to inter-stage connections. Our work effectively improved the model performance through simple improvements to the previous connection method. Experiments on three open-source hand pose datasets validated the effectiveness of the proposed model on hand pose estimation. For the finger tapping dataset we built, our model also achieved good results in predicting hand keypoint position and distance, velocity, and acceleration of the relative movement between two fingertips. The data collection of the PAKH dataset followed the test instructions and considered different hand postures. The performance of the model on this dataset can be seen as a reflection of that in practical applications. Based on these exciting results, we believe that our method is feasible in the finger tapping test and potentially other hand movement experiments.

### 5.2. Model Performance on Different Datasets

Our model achieved lower errors on our PAKH dataset than on the three open-source datasets: NYU, ICVL, and MSRA. This can be explained by the differences in the variety of hand postures in different datasets. Our PAKH dataset is built based on the finger tapping test, which is a cyclic motion. Compared to the open-source datasets that contain many different hand postures, the PAKH dataset has more homogeneous data. Deep learning models typically perform better on homogeneous data, and our model thus achieved lower errors on the PAKH dataset. This result led us to believe that when being implemented on other tests characterized by cyclic motion, our method can also achieve better performance than on the open-source datasets.

### 5.3. Limitations and Future Work

Despite promising results on the PAKH dataset, our research has several limitations. First, our dataset only contains data from healthy subjects. However, the movement patterns of healthy subjects differ from those of Parkinson’s patients. Because of neurological disorders, the hand movements of Parkinson’s patients are often characterized by tremors, rhythmic shaking, and bradykinesia. These movements are less regular and, therefore, more difficult to predict. Whether our model can work well on such movements remains to be tested. Second, when collecting the PAKH dataset, we fixed the position of the camera and restricted the hand movements of the participants within a certain area. Although we augmented our dataset by geometric transformations of the original images, the augmented datasets did not fully cover all the situations in practical applications. Changes in the relative position of the camera and the hands of participants and the surroundings of the hands of participants could potentially affect the prediction results. To ensure that our method can be applied to practical scenarios, we need a larger dataset to validate it. Third, the finger tapping test is only one of the many hand movement tests. Although our method performed well on the finger tapping test, it is hard to say whether it will perform well on other tests. Further experiments and analysis on other hand movement tests are needed to test the capabilities of the proposed model on hand movement recognition.

### 5.4. Future Prospective

Compared to traditional sensor-based HPE, deep learning methods are less expensive, easier to deploy, and can be performed remotely. Our model achieved state-of-the-art performance among deep learning methods. It may still fall short in accuracy compared to sensor-based methods, but for a movement with a maximum of ten centimeters for most subjects, we consider an error of less than 3 mm to be acceptable. In the future, with the increasing computing power of personal computers and the development of deep learning models and depth cameras, deep learning models will become more efficient and accurate in hand pose estimation and can thus better assist physicians in diagnosing and rehabilitating hand-related diseases. Moreover, the low errors achieved by our model on kinematic indicators important for diagnosis prompt us to believe it is promising to use deep learning to build an efficient and accurate test-to-diagnosis pipeline in the future. At a time when epidemics are prevalent, there is reason to believe that such diagnostic methods that can be performed remotely will become mainstream in the future.

## 6. Conclusions

We propose a depth-image based shifted attention regression network (SARN) for convenient and accurate 3D hand pose estimation. The proposed method uses a shifted attention heatmap to weight the predictions of different pixels to obtain the hand keypoint positions. This shifted attention heatmap can fully exploit the spatial correlations between pixels and hand keypoints by leveraging the information in both spatial closeness heatmap and geometry closeness heatmap. Experiment results show that SARN achieved state-of-the-art performance on three open-source 3D hand pose datasets: NYU, ICVL, and MSRA, with 7.32 mm, 5.91 mm, and 7.17 mm of all-keypoint mean error, respectively. This demonstrates the effectiveness of the proposed method. The ablation study validates the validity of each design. To test the feasibility of SARN in hand movement recognition, we constructed a hand movement dataset of 26K depth images based on a finger tapping test. The proposed method achieved an all-keypoint mean error of 2.99 mm and comparable performance on three task-specific metrics: the distance, velocity, and acceleration of the relative motion of the two fingertips. The success of the proposed method on this dataset validates its potential for applications in hand movement recognition.

## Figures and Tables

**Table 1:** The number of frames for each gesture in PAKH in training and testing.

Dataset	Subject	Gesture 1	Gesture 2	Gesture 3	Gesture 4	Total
Training	1	462	375	359	407	1603
	2	472	399	399	402	1672
	3	321	370	233	292	1216
	4	360	333	302	305	1300
	5	469	426	367	408	1670
	6	299	269	333	340	1241
	7	325	316	309	326	1276
	8	335	325	276	301	1237
	9	357	358	415	404	1534
	10	542	399	383	374	1698
	11	564	515	468	514	2061
	12	415	447	401	423	1686
	13	482	462	433	453	1830
	Total	5403	4994	4678	4949	20,024
Test	14	343	310	351	333	1337
	15	357	319	283	310	1269
	16	413	404	441	401	1659
	17	511	459	460	439	1869
	Total	1624	1492	1535	1483	6134

**Table 2:** Comparison with state-of-the-art methods on three public datasets: NYU, ICVL, MSRA.

Methods	NYU	ICVL	MSRA
Ren-9x6x6 [19]	12.69	7.31	9.79
Pose-REN [15]	11.81	6.79	8.65
DenseReg [32]	10.2	7.3	**7.23**
3DCNN [41]	14.1	-	9.58
V2V-PoseNet [28]	8.42	6.28	7.59
SHPR-Net [20]	10.78	7.22	7.76
HandPointNet [26]	10.54	6.94	8.5
Point-to-Point [38]	9.1	6.3	7.7
CrossInfoNet [42]	10.08	6.73	7.86
TriHorn-Net [17]	7.68	**5.73**	**7.13**
Ours	**7.32**	**5.91**	**7.17**

**Table 3:** Comparison of different heatmaps.

Methods	Mean Error (mm)
Spatial	7.41
Geometry	7.68
Shifted (shared weights)	7.36
Shifted (stage-wise weights)	**7.32**

**Table 4:** Comparison of different backbone networks.

Methods	Params	Mean Error (mm)
ResNet-18	15.23M	8.03
ResNet-34	25.49M	7.86
ResNet-50	34.01M	7.69
Hourglass (one stage)	4.58M	7.84
SE-Hourglass (one stage)	4.70M	7.78
Hourglass (two stages)	8.74M	7.53
SE-Hourglass (two stages)	8.98M	**7.49**

**Table 5:** Comparison of different input aggregation methods.

Methods	Mean Error (mm)
no processing	7.43
Conv	7.49
Conv+$I_{1}$	7.38
Soft (Ours)	**7.32**

**Table 6:** Performance on PAKH Dataset (mean ± std).

Methods	Pos Err	Dis Err	Vel Err	Acc Err
SARN	2.99 ± 2.33	2.98 ± 2.97	3.32 ± 3.32	5.65 ± 5.52

## Data Availability

The public datasets are available at: NYU: https://jonathantompson.github.io/NYU_Hand_Pose_Dataset.htm (accessed on 2 September 2022), ICVL: https://labicvl.github.io/hand.html (accessed on 15 August 2022), and MSRA: https://jimmysuen.github.io/ (accessed on 22 September 2022). Our collected data used for this study will be provided on demand.

## Abbreviations

The following abbreviations are used in this manuscript:

HPE	Hand pose estimation
SARN	Shifted attention regression network
AR	Augmented reality
VR	Virtual reality
HCI	Human-computer interaction
CNN	Convolutional neural network
PIP	Proximal interphalangea
MCP	Metacarpophalangeal
SE	Squeeze-and-excitation
